# Comparison of the Effects of Target-Controlled Infusion of Propofol and Sevoflurane as Maintenance of Anesthesia on Hemodynamic Profile in Kidney Transplantation

**DOI:** 10.1155/2019/5629371

**Published:** 2019-11-29

**Authors:** Dita Aditianingsih, Besthadi Sukmono, Tjues A. Agung, Willy Y. Kartolo, Erika S. Adiwongso, Chaidir A. Mochtar

**Affiliations:** ^1^Department of Anesthesiology and Intensive Care, Faculty of Medicine Universitas Indonesia, Cipto Mangunkusumo General Hospital, Diponegoro Street No. 71, DKI Jakarta 10430, Indonesia; ^2^Department of Urology, Faculty of Medicine Universitas Indonesia, Cipto Mangunkusumo General Hospital, Diponegoro Street No. 71, DKI Jakarta 10430, Indonesia

## Abstract

**Background:**

Target-controlled infusion (TCI) propofol and sevoflurane are common agents for general anesthesia, including for kidney transplantation procedure. This study compared the effect of TCI propofol and sevoflurane on intraoperative hemodynamic profile in kidney transplant patients.

**Methods:**

A single-blinded prospective study was performed in 46 kidney transplant recipients who were randomized into receiving TCI propofol or sevoflurane as anesthetics maintenance. Hemodynamic parameters such as mean arterial pressure (MAP), cardiac index (CI), stroke volume index (SVI), and systemic vascular resistance index (SVRI) were measured at baseline before induction, postintubation, first surgical incision, every 15 minutes after the first incision, reperfusion, and 15 minutes after reperfusion. Data were analyzed using unpaired *t*-test, paired *t*-test, and general linear model.

**Results:**

Intraoperative MAP, CI, SVI, and SVRI changes were similar in both groups (*p* = 0.480, 0.216, 0.086, and 0.054). In comparison to the baseline value, TCI propofol and sevoflurane groups showed significant reductions of MAP at postintubation (*p*=0.010; *p* < 0.001) and during the first surgical incision (*p*=0.009; *p* < 0.001); significant reduction of CI at postintubation (*p*=0.003; *p* < 0.001) and during the first surgical incision (*p* < 0.001; *p* < 0.001); significant reduction of SVI at postintubation (*p*=0.013; *p*=0.008), during the first surgical incision (*p*=0.008; *p*=0.003), and 15 minutes after reperfusion (*p*=0.010; *p*=0.005); and significant increasing of SVRI during the first surgical incision (*p*=0.007; *p*=0.005). The TCI propofol group showed significantly lower SVRI compared to the sevoflurane group postintubation (*p*=0.029) and during the first surgical incision (*p*=0.026).

**Conclusion:**

Intraoperative hemodynamic profile was similar between the TCI propofol and sevoflurane group during kidney transplant surgery. The TCI propofol group had higher CI and SVI but showed significantly lower SVRI as compared to the sevoflurane group. The incidence of postanesthesia agitation, postoperative outcome, and complication were not significantly different between the two groups.

## 1. Introduction

Kidney transplantation is one of the preferred treatments for end-stage renal disease (ESRD) with the increasing rate of success and high quality of life outcome, compared to patients who did not undergo kidney transplantation [1–4]. Chronic kidney disease (CKD) is a clinical syndrome related to the metabolic and systemic disorder caused by gradual homeostatic and kidney excretion decline as a result of irreversible kidney damage. The failing of kidney function will have negative effects on many other organ systems [4–7]. The cardiovascular disorder evolves at the early stage of CKD and often shows as the coronary and brain ischemic vascular diseases. Systemic hypertension, dilated cardiomyopathy, and concentric ventricular hypertrophy are caused by an increase of cardiac output in response to increase in intravascular volume, pressure overload, anemia, and increased vascular resistance due to a high level of renin-angiotensin released by the damaged kidney. Arteriosclerosis is a vascular disorder in CKD which can be quickly developed by having diabetes and dyslipidemia [5, 6, 8].

Cardiovascular disturbance is the main cause of morbidity and mortality in CKD patients. It almost reached 50% of all mortality causes in CKD with dialysis and the highest cause of perioperative transplant deterioration, which could lead to delayed graft function [8, 9]. Patient comorbid factor in addition to anesthetic agent effect is the challenge for an anesthesiologist to maintain the intraoperative hemodynamic stability during transplantation surgery [10]. Hemodynamic parameters including cardiac output (CO), stroke volume (SV), and systemic vascular resistance (SVR), or which indexes according to body surface area (BSA) and mean arterial pressure (MAP) are continually recorded through a semi-invasive monitor during and after surgery. Intraoperative monitoring and using an appropriate anesthetic regimen that assures hemodynamic stability in kidney transplantation are important to prevent delayed graft function of the new kidney and decrease the risk of postoperative dialysis [5, 8, 11]. The use of drugs with low metabolism, short half-life, and extrarenal clearance should be the choice to allow a rapid emergence with early cognitive and psychomotor function recovery [12].

The general anesthesia with an inhalational agent, intravenous (IV), or combination are anesthesia techniques routinely used in kidney transplantation [3, 13]. Sevoflurane is the volatile anesthetic agent commonly used for its nonpungent and less irritating characteristics [2]. Sevoflurane is a fast onset anesthesia inhalation agent and is more commonly used in kidney transplantation compared to isoflurane and desflurane [2]. Propofol is a short-acting IV anesthetic agent, also used during transplantation procedures. Pharmacokinetic models for target-controlled infusion (TCI) have facilitated the gradual and controlled administration of propofol, mainly through the total intravenous anesthesia (TIVA) technique [14]. The cardioprotective effects between volatile agents and propofol differ in their mechanism [15].

Depending on the timing of administration, anesthetic agents such as sevoflurane, isoflurane, and to a lesser extent propofol may induce biochemical changes that may attenuate ischemia-reperfusion injury (IRI). Conditioning effects of volatile anesthetics on the heart and kidney were examined in vitro, in animal models, and in randomized controlled clinical trials. The results remain conflicting since the translation of protective effects by volatile anesthetics from experimental models of IRI into clinical study does not significantly improve patient's morbidity and long-term outcome compared with a propofol-based anesthetic technique. Only several small sample size studies that lack blinding design showed sevoflurane was associated with a lower incidence of late adverse cardiac events. Besides the mode of administration of the anesthetic agent, the study of anesthetic-induced conditioning in clinical setting depends on many interacting factors and disease states as the confoundings, especially in noncardiac surgery [15–18]. Sevoflurane and propofol have some cardiovascular side effects, mainly negative inotropic effect from the volatile agent and predominantly vasodilatory effect of propofol [14, 19–22]. Some reports showed propofol has lower incident of hangover and emergence agitation incidences in contrast to sevoflurane [23]. Besides the clinical safety-efficacy and pharmacokinetic profile for a particular surgical procedure such as kidney transplant, the selection of an anesthetic agent should be considered for its feasibility and cost-effectiveness [24].

Our study aimed to compare the effects between TCI of propofol and sevoflurane on intraoperative hemodynamic profile in the kidney transplant recipient. We hypothesized that TCI of propofol resulted in more stable intraoperative hemodynamics in the kidney transplant recipient than sevoflurane as the anesthesia maintenance agent. The hemodynamic parameters recorded as our primary outcomes were intraoperative cardiac index (CI) and MAP. The secondary outcomes were stroke volume index (SVI) and systemic vascular resistance index (SVRI). Other variables and outcomes such as intraoperative fentanyl consumption, minimum-maximum range of vasoactive dosage, postanesthesia agitation, urine production, delayed graft function, and the expenditure of propofol and sevoflurane as the anesthesia maintenance were analyzed.

## 2. Materials and Methods

### 2.1. Ethical Consideration

This prospective randomized open-label controlled study was approved by the Ethics and Research Committee of Universitas Indonesia (approval no. 600/UN2.F1/ETIK/2017) and was registered on July 11, 2017, in clinicaltrials.gov (NCT03214653).

### 2.2. Sample Size and Patient Enrolment

Sample size calculation was based on the hypothesis that TCI propofol provides stability in intraoperative hemodynamic as a single agent. According to previous studies [25–27], the mean decrease in MAP after induction was 23 (SD = 8) and CI was 2.6 (SD = 0.19) in TCI propofol groups, and the mean decrease in MAP after induction was 28 (SD = 5) in the sevoflurane group, and CI was 2.0 (SD = 1.03) in the sevoflurane group. A difference of 20% in CI and MAP between the 2 groups after induction was considered as clinically relevant. With a common standard deviation of 6, a sample size of 23-24 patients in each group was determined with a statistical power of 0.8 and a type-1 error of 0.05 using the sample size calculator (http://www.stat.ubc.ca). This study recruited a total of 50 patients to allow 10% dropouts. All patients provided written informed consent prior to participation. The inclusion criteria were kidney transplant recipient, age between 20 and 75 years, body mass index (BMI) 18–35 kg/m^2^, and Charlson's Comorbidity Index (CCI) score 1–4. Exclusion criteria were blood loss >15 mL/kg and hyperacute graft rejection with hemodynamic instability. Randomization was performed by an attending anesthetist through a sealed envelope. Patients were equally assigned into the TCI propofol or sevoflurane group.

### 2.3. Anesthesia Procedure

All patients received ranitidine (50 mg IV), ondansetron (4 mg IV), methylprednisolone (500 mg IV), and cefoperazone (1 g IV) prophylactic antibiotics in the preparation room. All patients had electrocardiography (ECG), oxygen saturation, noninvasive blood pressure monitoring, and bispectral index (BIS). The patients received premedications of midazolam (2 mg IV) and fentanyl (1 mcg/kg IV), and then the arterial cannula at the radial artery and central vein catheter at the subclavian or internal jugular vein were inserted with local anesthetic and ultrasonography (USG) guidance. Baseline hemodynamic parameter was recorded through the pulse contour analysis method using a semi-invasive monitor EV1000™ (Edwards Lifesciences, Irvine, California) before anesthesia induction. The anesthesia induction was performed with fentanyl (3 mcg/kg IV) and propofol (1–1.5 mg/kg IV). The endotracheal tube (ETT) intubation was facilitated with atracurium (0.5 mg/kg IV). The ventilator was set to volume control with tidal volume 8–10 ml/kg, PEEP 5 cmH_2_O, and FiO_2_ 30–50% and breathing frequency was adjusted with ETCO_2_ target of 35–45 mmHg and oxygen compressed air ratio of 40 : 60. The anesthesia was maintained using sevoflurane for the control group or TCI propofol using the Schnider model by entering age, gender, height, and total body weight (TBW) to calculate lean body weight (1.1 × TBW−128 × (weight/height)^2^ and effect-site concentration target (*C*_E_) setting for the intervention group, according to the randomization along with the BIS target of 45–55. Intraoperative analgesia was maintained by continuous fentanyl (1 mcg/kg/hour IV), with extra 1 mcg/kg IV boluses if the heart rate increases >20%. Muscle relaxant atracurium (0.5 mg/kg/hour) was administered continuously with a target of train of four ratio ≤25% during surgery. The MAP was maintained at 70–90 mmHg with vasoactive and inotropic agents as necessary. Mannitol 20% (0.5 mg/kg IV) was administered during the cold ischemic time, and furosemide (1 mg/kg IV) boluses were given during the warm ischemic time before the reperfusion. All patients had an epidural catheter inserted after the completion of surgery for postoperative pain management.

### 2.4. Sample Collection and Statistical Analysis

The artery cannula was inserted at the radial or brachial artery and connected to a semi-invasive monitor EV1000™ to measure the CI, SVI, and SVRI continuously by pulse contour analysis [20, 28, 29]. The value of CI, MAP, SVI, and SVRI was recorded before induction as baseline (T0), at 5 minutes postintubation (T1), first surgical incision (T2), every 15 minutes after incision (T3–T12), reperfusion (T13), and another 15 minutes after reperfusion as 15 minutes after reperfusion (T14). Intraoperative fentanyl consumption, minimum-maximum vasoactive dosage, urine production, the incidence of postanesthesia agitation, delayed graft function, and the expenditure of propofol and sevoflurane as the anesthesia maintenance were analyzed. The postanesthesia agitation was assessed during emergence and after extubation using Riker sedation-agitation whether the patients were calm, awakening easily, followed the instructions, anxious but calm with verbal commands (scale 3–5), or uncooperative such as not calm despite the verbal commands, bit or dragged the ETT, raised bedside bars, tried to remove catheter, attacked people, and showed struggles [30]. Delayed graft function was evaluated if there was an increase in serum creatinine, oliguria, and the requirement for dialysis in the first postoperative week.

Categorical data were presented in total (%) and analyzed with chi-square test. Numerical data were analyzed with unpaired *t*-test or Mann–Whitney test, paired *t*-test within each group, and general linear model (GLM) between two groups, with *p* < 0.05 considered as statistically significant, using Statistical Package for Social Scientist (SPSS) version 20. Numerical normal-distributed data were presented in mean ± standard deviation or median (minimum-maximum) for abnormal-distributed data.

## 3. Results

### 3.1. Research Subject Characteristics

**Figure 1:**
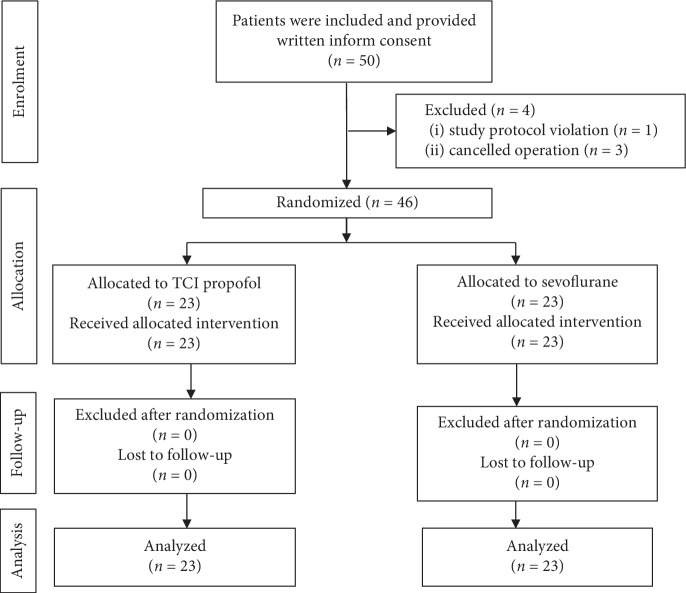
The flow diagram of the study.

This study enrolled 50 kidney transplant recipients from July to December 2017, who provided the written consent to participate. There was a total of 4 patients excluded from the study: 2 patients due to the donor problem, 1 patient due to acute lung edema before anesthesia induction, and 1 patient due to study protocol violation of artery and central venous line insertion after anesthesia induction. Therefore, 46 patients were equally randomized to two groups and included in final data collection and analysis. The CONSORT flow diagram is presented in Figure 1.

**Figure 2:**
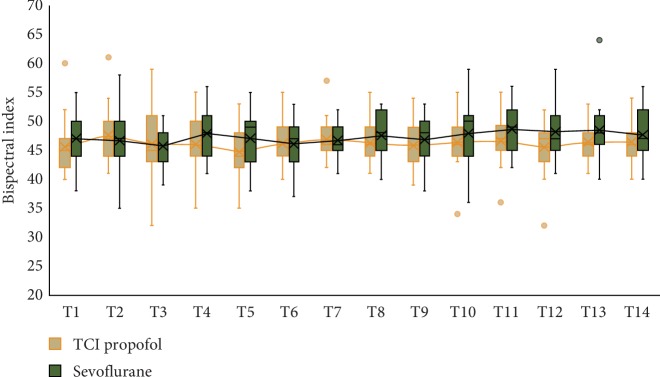
Intraoperative bispectral index value. Time points are as follows: postintubation (T1), first surgical incision (T2), every 15 minutes after the first incision (T3–T12), reperfusion (T13), and 15 minutes after reperfusion (T14).

There were no differences regarding subject baseline characteristics between the TCI propofol and sevoflurane group. The most common comorbidities were hypertension and diabetes mellitus (Table 1).  Table 2 shows a significant difference in the maximum dobutamine dosage 10 mcg/kg/min in the sevoflurane group (*p*=0.031) compared with the TCI propofol group. The average BIS value was not significantly different (*p*=0.540) between using sevoflurane at 0.7 (0.6–0.9) MAC and the TCI propofol Schnider model with effect-site concentration target (*C*_E_) 0.5–4.7 mcg/mL (Figure 2).

## 4. Intervention Result

**Figure 3:**
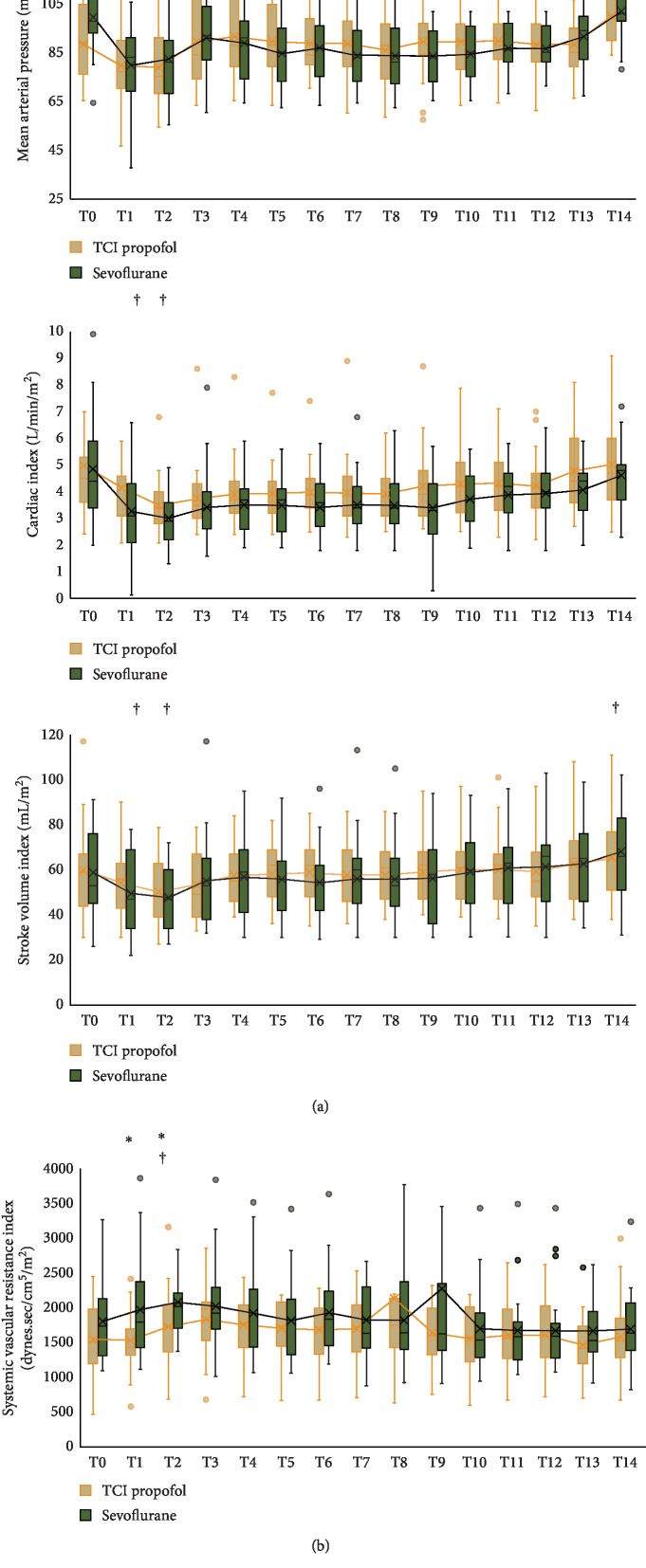
Intraoperative mean arterial pressure, cardiac index, stroke volume index, and systemic vascular resistance index. Time points are as follows: baseline (T0), postintubation (T1), first surgical incision (T2), every 15 minutes after first incision (T3–T12), reperfusion (T13), and 15 minutes after reperfusion (T14). ^∗^Unpaired t‐test for comparison between groups; † Paired t‐test for comparison with baseline; *p* < 0.005 is significant.

Figure 3 summarizes intraoperative MAP, CI, and SVI at specified time points (details of data are available in Supplementary [Supplementary-material supplementary-material-1]). The mean baseline MAP value in the TCI propofol group was lower compared to the sevoflurane group (*p*=0.011). In comparison with their baseline value, there were significant reductions of MAP postintubation (T1) and during the first surgical incision (T2) in the TCI propofol group (T1: 80.60 ± 18.22 vs. 89.47 ± 14.16 mmHg, *p*=0.010; T2: 79.78 ± 15.90 vs. 89.47 ± 14.16 mmHg; *p*=0.009) and in the sevoflurane group (T1: 80.73 ± 16.91 vs. 100.78 ± 14.83 mmHg, *p*=0.001; T2: 83.30 ± 18.56 vs. 100.78 ± 14.83 mmHg; *p* < 0.001). The MAP at 15 minutes after reperfusion (T14) was significantly higher compared to the baseline value in the TCI propofol group (101.08 ± 11.82 vs. 89.47 ± 14.16 mmHg, *p* < 0.001) but not in the sevoflurane group.

There was significant reduction of CI postintubation (T1) and during the first surgical incision (T2) compared to baseline in the TCI propofol group (T1: 3.89 ± 1.43 vs. 4.55 ± 1.50 L/min/m^2^, *p*=0.003; T2: 3.35 ± 1.33 vs. 4.55 ± 1.50 L/min/m^2^, *p* < 0.001) and in the sevoflurane group (T1: 2.78 ± 2.12 vs. 4.48 ± 1.50 L/min/m^2^, *p*=0.001; T2: 2.84 ± 1.41 vs. 4.48 ± 1.50 L/min/m^2^, *p* < 0.001). There was significant reduction of SVI postintubation (T1) and during the first surgical incision (T2) compared to baseline in the TCI propofol group (T1: 54.35 ± 15.40 vs. 59.91 ± 19.51 mL/m^2^, *p*=0.013; T2: 50.26 ± 14.33 vs. 59.91 ± 19.51 mL/m, *p*=0.008) and in the sevoflurane group (T1: 49.56 ± 17,76 vs. 58.86 ± 18.13 mL/m^2^, *p*=0.008; T2: 47.73 ± 13.80 vs. 58.86 ± 18.13 mL/m^2^, *p*=0.003). The SVI at 15 minutes after reperfusion (T14) was significantly higher compared to the baseline value both in the TCI propofol group (65.13 ± 18.65 vs 59.91 ± 19.51 mL/m^2^, *p*=0.010) and in the sevoflurane group (68.08 ± 19.30 vs. 58.86 ± 18.13 51 mL/m^2^, *p*=0.005) due to fluids and vasoactive administration.

The SVRI was significantly increased during the first surgical incision (T2) compared to baseline in the TCI propofol group (1718.21 ± 531.61 vs. 1541.04 ± 514.33 dynes.sec/cm^5^/m^2^, *p*=0.007) and in the sevoflurane group (2070.34 ± 617.98 vs. 1797.47 ± 563.43 dynes.sec/cm^5^/m^2^, *p*=0.005). TCI propofol groups showed significant lower SVRI compared to sevoflurane group postintubation (T1) (1521.17 ± 466.44 vs. 1964.86 ± 829.14 dynes.sec/cm^5^/m^2^, *p*=0.029) and during the first surgical incision (T2) (1718.21 ± 531.61 vs. 2070.34 ± 617.98 dynes.sec/cm^5^/m^2^, *p*=0.026). There were no significant trend differences between the two groups in MAP (*p*=0.480), CI (*p*=0.216), SVI (*p*=0.086), and SVRI (*p*=0.054) using GLM analysis.

The incidence of postanesthesia agitation was lower in the TCI propofol group (5/23 vs. 7/23; *p*=0.502), with odds ratio 0.635 (0.168–2.402) compared with the sevoflurane group. The expenditure of using TCI propofol as the anesthesia maintenance was higher than sevoflurane (IDR 741,651.00 vs. IDR 399,944.00, *p* < 0.001).  Table 3 shows the postoperative outcome, and complications such as delayed graft function, return to dialysis after transplant failure, posttransplant infection, and mortality in 1 year follow-up were not significantly different. One patient died because of infection, 1 patient died due to acute coronary syndrome, and 2 patients died having multiorgan failure after graft rejection.

## 5. Discussion

### 5.1. Research Subject Characteristic

Nowadays, the kidney transplant therapy is considered a promising and saving therapy, yet, having some hemodynamic drawbacks. Mortality as the biggest risk that threatens the kidney transplantation patients during the first year is related to age factors: 2% of age group 18–34 years, 3% of age group 35–49 years, and 6.8% of age group ≥50 years [4, 7, 8, 31]. Preanesthesia evaluation including cardiac function examination is essential to diagnose acute or chronic coronary artery disease, determine the functional status of the patient, and optimize the therapy before undergoing kidney transplantation [5].

Propofol sedation effect arises through increase in bonding affinity of gamma-aminobutyric acid (GABA) neurotransmitter to GABA_A_ at the central nervous system. The modulation at hippocampus will inhibit the release of acetylcholine from the hippocampus and prefrontal cortex [21]. Propofol can be used through the TCI technique, where the dosage can be adjusted according to plasma-site or effect-site concentration target. During TCI technique, the bispectral index can be useful in some patients, for a guidance of anesthesia depth. The recommended range value of 40–60 for BIS monitoring during the maintenance phase of general anesthesia needs to be tailored depending on the technique, procedure, and patient conditions [19]. The dosage of TCI propofol and sevoflurane in this research was adjusted to the BIS target of 45–50, with 1–1.2 L/min mixed fresh gas flow of oxygen and air.

With the same amount of intraoperative fentanyl usage, there was a trend of lower BIS value in the sevoflurane group with 0.7 MAC of volatile maintenance compared to the TCI propofol group. The recorded BIS value is not always precisely correlated with the level of anesthesia depth since the BIS value can be influenced by patient conditions or the effect of anesthetic agents. Paradoxical awakening reaction due to an increase of alpha waves in the EEG is denoted by the increase in the BIS value when the inspired fraction of inhalational agent was increased, and then the BIS value returned to baseline levels after reducing the concentration of the inhalational agent. The loss of consciousness with a higher BIS value can be associated with the addition of opioid to the lower concentration of propofol. The hypnotic effects of propofol are increased by opioids, but the BIS value does not show this effect. Elevated electromyographic (EMG) activity can increase the BIS value, and the subsequent administration of neuromuscular blockers (NMBs) will reduce it. A lower BIS value accompanied by low blood pressure at low MAC of volatile indicates a sensitivity to anesthesia [32].

### 5.2. Hemodynamic Parameters Analysis

There are a limited number of randomized clinical trial and available data on the effect of sevoflurane and TCI propofol on hemodynamic profile during kidney transplantation. Most of the studies compared the effects of sevoflurane and propofol on postoperative analgesia and the incidence of postoperative nausea and vomiting (PONV). Only 14 randomized controlled studies compared the effect of sevoflurane and propofol as anesthesia maintenance agents on intraoperative hemodynamic, but none of them were performed on transplant surgery [33, 34]. The MAP, CI, SVI, and SVRI values describe the heart contractility and preload and afterload changes [20, 28, 29]. Our study showed the patients undergoing sevoflurane or TCI propofol anesthesia had similar MAP, CI, SVI, and SVRI during kidney transplant surgery. The CI tended to be more stable maintained in the TCI propofol group; however, SVRI was significantly lower in postintubation and during the first surgical incision in the TCI propofol group compared to the sevoflurane group. Our results showed the MAP was similar between the two groups, could be due to the preserved CI in the TCI propofol group and preserved SVRI in the sevoflurane group. Increasing concentrations of propofol within the therapeutic range resulted in decreasing arterial and venous resistance in a similar mechanism. The decreasing vascular resistance without a change in CI can be a result of the balance between a decrease in effective or stressed volume that is determined by the mean systemic filling pressure and a decrease in resistance for venous return therefore preserved the SVI and CI [35]. Sevoflurane induces myocardial depression at 1.0 or higher MAC, resulting in decreased CI, and produces vasodilatation at or higher than 1.5 MAC or 3 vol % that reduces SVRI [36, 37], while our patients only received 0.7 (0.6–0.9) MAC or 1.4 (1.2–1.8) vol % in the sevoflurane group.

There were significant reductions of MAP, CI, and SVI on both groups after anesthesia induction and during the first incision. Those findings were similar to Robba et al. study that compared the propofol-based anesthesia and sevoflurane-based anesthesia in cervical spine surgery and found significant reduction of MAP on both groups after anesthesia induction, in which the propofol groups had lower mean MAP than the sevoflurane group after induction and intubation [25]. In VAPOR-1 study, the kidney transplant patients receiving sevoflurane as the maintenance agent needed boluses of ephedrine more frequent than patients anesthetized with propofol especially after induction although no prolonged hypotensive periods were observed [18].

The highest maximum norepinephrine dosage was seen in the TCI propofol group to encounter the lower mean baseline MAP value in the TCI propofol group compared to the sevoflurane group. The patients in the sevoflurane group might have more severe hypertension that resulted in a higher mean baseline MAP value and receiving more *α*-agonist considering it is not usually used as the first-line antihypertension drug. Vasopressor is used to increase the decreasing SVRI and CI due to vasodilatation after the anesthesia induction [38]. The maximum dobutamine dosage was significantly higher in the sevoflurane group that could be because more *β*-blocker and *α*-agonist antihypertension agents were used in this group. Dobutamine increases SV and CI because of its inotropic effect that contributes to maintaining the MAP [38].

The earlier study by Modesti et al. showed the trend of hemodynamic parameters (heart rate, systolic-diastolic blood pressure, and MAP) were not statistically different between using TIVA propofol 6 mg/kg/hour and remifentanil 0.1–0.5 *μ*g/kg/hour or balanced anesthesia with isoflurane (0.8–1 MAC) and intermittent fentanyl boluses in kidney transplant surgery. Although their TIVA regimens were not delivered by TCI methods, the results were similar to our study that showed a stable hemodynamic and had the potential to be an alternative to the balanced anesthesia. The TIVA group presented a faster recovery but worse pain control within the first postoperative hour compared to balanced anesthesia using an inhalational agent. Remifentanil undergoes ester hydrolysis resulted in rapid metabolism, short duration of action; however, it may influence the occurrence of postoperative hyperalgesia compared to fentanyl [12].

The different populations might be the cause of the different results with Shimonov et al. who found sevoflurane led to more hemodynamic instability as compared to propofol in laparoscopic radiofrequency tumor ablation [39]. Most of our kidney transplant recipients had cardiovascular comorbidities such as hypertension, history of coronary artery disease, or heart failure. Those conditions affected hemodynamic changes critically during induction, intubation, and reperfusion under anesthesia. Sevoflurane attenuates arterial baroreflex function that may affect the hemodynamic stability during anesthesia [40]. Sevoflurane develops its negative inotropic effect from the disruption of calcium (Ca^2+^) in the cardiac myocyte which leads to a decrease in the available amount of intracellular Ca^2+^. The cardiac effects caused by sevoflurane are dose-dependent, accentuating in comorbid conditions like contractility dysfunction or electric conduction disorder [14, 41]. Propofol has a cardiovascular effect such as the decrease of preload and arterial blood pressure because of sympathetic vasoconstriction inhibition that leads to the decrease in CO through inhibition of intracellular Ca^2+^ mobilization in the smooth muscle [19, 21, 22]. Although, mostly, our ESRD patients have above normal SVRI, their cardiovascular hemodynamics are susceptible to be depressed by anesthesia agents such as sevoflurane and propofol [20, 28, 29].

After reperfusion, MAP in the TCI propofol group and SVI in both groups were significantly higher than their baseline value. That could be due to fluids and vasoactive administration, with the maximum dosage of dobutamine requirement higher in the sevoflurane group compared to the TCI propofol group. Those results were similar to the study on propofol-based vs. sevoflurane-based anesthesia conducted by Nieuwenhuijs-Moeke et al. who stated the average MAP during transplantation procedure was higher in the propofol group compared with the sevoflurane group. Hemodynamic profiles of both groups were comparable; however, patients in the sevoflurane group more frequently received a bolus of ephedrine compared with patients in the propofol group [18]. Sevoflurane affects the hemodynamic stability through attenuating arterial baroreceptor function and delay the changes of systolic blood pressure in response to baroreflex stimulation [14]. Propofol-based anesthesia maintains the baroreceptor reflex sensitivity and CI, but it has effects in reducing systolic and diastolic arterial pressure by lowering SVRI and afterload [42]. Propofol decreases SVRI without significant changes in CI due to the balance between the decrease of mean systemic filling pressure and resistance of venous return [35].

The uncalibrated pulse contour method consists of pressure measurement and the arterial waveform and no external calibration is required. This system determines CO from the arterial waveform that corresponds to the vascular tone and is calculated using a pulse rate, BSA, aortic compliance, MAP, and the arterial pressure over a certain time that forms the arterial pressure curve. The SV was calculated from a pressure measurement that is converted to volume measurement. Deriving a flow from a pressure parameter requires concise information of the pressure-volume relation in the arterial system and especially the aorta that incorporates arterial impedance, arterial compliance, and systemic vascular resistance. Converting a pressure measurement into a volume parameter is prone to inaccuracy, especially in patients with changes in aortic impedance induced by changes in the SVR or with low CO. Location of arterial line inserted at the radial artery, change in systemic vascular resistance, and aortic impedance induced by catecholamines influence the calibration factor. However, the norepinephrine and dobutamine dosage in our study period were considered low to moderate for influencing the calibration factor of the arterial pulse curve.

### 5.3. Extra Analysis Data

We recorded postanesthesia agitation during extubation and found the agitation incidence was higher in the sevoflurane group compared to the TCI propofol group although not significantly different. This finding is in concordance to the 2014 meta-analysis from Cochrane that stated the agitation risk was lower when using propofol as the maintenance agent compared to sevoflurane (RR 0.35, CI 95% 0.25–0.51), and propofol reduces agitation risk when used only during the maintenance phase of anesthesia after sevoflurane induction (RR 0.59, CI 95% 0.46–0.76) [43]. In anesthesia with sevoflurane, the difference in recovery speed within the nervous system increases the sensitivity to stimulation from the surrounding environment, creating a state of functional dissociation. The emergence agitation may occur from the changes and relationship of GABA_A_ receptors in the central nervous system and decrease inhibitory signals from the globus pallidus interna and substantia nigra. The inability to suppress thalamocortical neurons and brain stem neurons remains the sedative effect in the early stages of emergence and the euphoria in the later stage. Compared to inhalation anesthetics, propofol has a lower incidence of PONV and has a lower occurrence of the hangover that could be related to the reduction in occurrence of emergence agitation [23]. Our study showed the average expenditure was higher in using TCI propofol for intraoperative anesthesia maintenance compared to using sevoflurane. A similar result was found in a study by Struys et al. who analyzed the expenditure of continuous propofol compared to sevoflurane as the maintenance agent with the guidance of BIS value in gynecologic surgery and stated that the expenditure of propofol was significantly higher than sevoflurane [29].

There were no significant differences in delayed graft function, dialysis after kidney transplant failure, and posttransplant infection between groups although nonsignificant higher mortality was seen in the TCI propofol group. Intraoperative hemodynamic stability is essential to maintain organ perfusion, while anesthetic conditioning such as sevoflurane and propofol has a protective effect to attenuate IRI and prevent delayed graft function [5, 8, 11]. However, our study sample was not designed to have enough power for detecting postoperative complications and outcome.

### 5.4. Research Limitation

Although the distribution was equal in both groups, the variety of age might affect the patient's vascular elasticity and pulse contour analysis hemodynamic measurement in this study. The research subjects had taken multidrug therapy which could affect the cardiovascular function; however, the results could represent the actual kidney transplant population. Our study did not include stroke volume variation analysis since the value could not be accurate in spontaneous breathing patients during the baseline measurement. The first-time usage and the duration of vasoactive administration were not recorded.

## 6. Conclusions

The intraoperative hemodynamic profile was similar between TCI propofol- and sevoflurane-based anesthesia during kidney transplant surgery. Using TCI propofol as anesthesia maintenance resulted in higher CI and SVI, but lower SVRI and MAP than using sevoflurane in kidney transplantation.

## Figures and Tables

**Table 1:** Patients' characteristics.

Variable		TCI propofol (*n* = 23)	Sevoflurane (*n* = 23)	*p* value
Sex	Male	18 (78.3%)	17 (73.9%)	1
	Female	5 (21.7%)	6 (26.1%)	
Age (years)		50.5 ± 14.4	50.2 ± 13.7	0.933
Body weight (kg)		67.6 ± 15.4	66.3 ± 12.9	0.759
Height (m)		1.65 ± 0.06	1.63 ± 0.09	0.530
Body mass index (kg/m^2^)		24.8 ± 4.9	24.7 ± 3.3	0.980
Body surface area (m^2^)		1.7 ± 0.2	1.7 ± 0.2	0.733
Type 2 diabetes		8 (34.8%)	10 (43.5%)	0.763
Coronary artery disease		5 (21.7%)	4 (17.4%)	1
Hypertension with				
Angiotensin receptor blocker		14 (60.9%)	14 (60.9%)	1
*β*-Blocker		8 (34.8%)	13 (56.5%)	0.236
Calcium channel blocker		19 (82.6%)	16 (69.6%)	0.489
Angiotensin-converting enzyme inhibitor		0 (0%)	2 (8.7%)	0.489
*α*-Agonist		1 (4.3%)	9 (39.1%)	0.012
Charlson's comorbidity index		3.0 (2.0–4.0)	3.0 (2.0–4.0)	1

Categorical variable presented in total (%). Numeric variable presented with mean (±standard deviation) or median (minimum-maximum). Data are analyzed using chi-square or unpaired *t*-test.

**Table 2:** Patients' perioperative data.

Variables		TCI propofol (*n* = 23)	Sevoflurane (*n* = 23)	*p* value
Surgery duration (hour)	>3-4	1 (4.3%)	4 (17.4%)	0.268
	>4-5	13 (56.5%)	13 (56.5%)	
	>5-6	9 (39.1%)	4 (17.4%)	
	6	0 (0%)	2 (8.7%)	
Anesthesia duration (hour)	>3-4	0 (0%)	0 (0%)	0.583
	>4-5	1 (4.3%)	1 (4.3%)	
	>5-6	9 (39.1%)	11 (47.8%)	
	>6	13 (56.5%)	11 (47.8%)	
Sevoflurane concentration (MAC)			0.7 (0.6–0.9)	
Total propofol usage (mg/hour)		1,200 (650–3,325)		
Total blood loss (ml)		75.0 (50–150)	100 (50–200)	0.134
Total intraoperative fluid (ml)		500 (150–1,000)	700 (400–1,000)	0.334
Minimum norepinephrine dosage (mcg/kg/min)		0.02 (0.01–0.05)	0.01 (0.01–0.05)	0.191
Maximum norepinephrine dosage (mcg/kg/min)		0.15 (0.05–0.5)	0.1 (0.05–0.4)	0.550
Minimum dobutamine dosage (mcg/kg/min)		0.1 (0.1–1)	0.1 (0.1–3)	0.079
Maximum dobutamine dosage (mcg/kg/min)		5 (0–5)	10 (0–10)	0.031^*∗*^
Intraoperative fentanyl usage (mcg/kg/hour)		1.1 ± 0.5	1.0 ± 0.4	0.490
Urine output postreperfusion (ml/hour)		382.6 ± 215.4	410.21 ± 346.88	0.817

Data are presented as total (%) or as geometric mean and confidence interval 95% (minimum-maximum), *p* < 0.05 is significant. Data are analyzed with chi-square, unpaired *t*-test, or Mann–Whitney. TCI, target-controlled infusion.

**Table 3:** Postoperative clinical outcome and complication.

Outcomes in 1-year follow-up	Sevoflurane group (*N* = 23)	TCI propofol group (*N* = 23)	*p* value
Delayed function graft	0	1	1.000
Posttransplant dialysis	0	1	1.000
Infection	0	1	1.000
Deceased	1	3	0.608

Data analyzed using Fisher's exact test. *p* value <0.05 considered significant.

## Data Availability

The data set used and/or analyzed in the current study are available from the corresponding author upon reasonable request.

## Supplementary Materials

Supplementary Materials Supplementary Table 1: intraoperative hemodynamic changes at specified time points.
