# Supplementary material for: Comparison of the Effects of Target-Controlled Infusion of Propofol and Sevoflurane as Maintenance of Anesthesia on Hemodynamic Profile in Kidney Transplantation
Source: Anesthesiol Res Pract. 2019 Nov 29;2019:5629371. doi: 10.1155/2019/5629371 (PMC6925739; doi:10.1155/2019/5629371)
Supplement: Supplementary Materials — Supplementary Table 1: intraoperative hemodynamic changes at specified time points. [file 5629371.f1.docx]

**Supplementary Table 1.** Intraoperative hemodynamic changes at specified time-points.

| **Timing** | **TCI Propofol**  **(n = 23)** | **Sevoflurane**  **(n = 23)** | **p value*** |
| --- | --- | --- | --- |
| **Mean arterial pressure (mmHg)** | | | |
| Baseline (T0) | 89.47 ± 14.16 | 100.78 ± 14.83 | 0.011* |
| Postintubation (T1) | 80.60 ± 18.22^†^ | 80.73 ± 16.91^†^ | 0.980 |
| First surgical incision (T2) | 79.78 ± 15,90^†^ | 83.30 ± 18,56^†^ | 0.493 |
| Reperfusion (T13) | 89.30 ± 13.11 | 92.52 ± 10.27 | 0.359 |
| 15 minutes after reperfusion (T14) | 101.08 ± 11.82^†^ | 103.13 ± 11.36 | 0.553 |
|  |  |  |  |
| **Cardiac index (L/min/m^2^)** | | | |
| Baseline (T0) | 4.55 ± 1.50 | 4.48 ± 1.50 | 0.897 |
| Postintubation (T1) | 3.89 ± 1.43^†^ | 2.78 ± 2.12^†^ | 0.062 |
| First surgical incision (T2) | 3.35 ± 1.33^†^ | 2.84 ± 1.41^†^ | 0.089 |
| Reperfusion (T13) | 4.53 ± 1.37 | 3.95 ± 1.29 | 0.113 |
| 15 minutes after reperfusion (T14) | 4.76 ± 1.39 | 4.45 ± 1.32 | 0.455 |
|  |  |  |  |
| **Stroke volume index (mL/m^2^)** | | | |
| Baseline (T0) | 59.91 ± 19.51 | 58.86 ± 18.13 | 0.845 |
| Postintubation (T1) | 54.35 ± 15.40^†^ | 49.56 ± 17.76^†^ | 0.234 |
| First surgical incision (T2) | 50.26 ± 14.33^†^ | 47.73 ± 13.80^†^ | 0.538 |
| Reperfusion (T13) | 62.52 ± 18.39 | 62.78 ± 18,10 | 0.974 |
| 15 minutes after reperfusion (T14) | 65.13 ± 18.65^†^ | 68.08 ± 19.30^†^ | 0.644 |
|  |  |  |  |
| **Systemic vascular resistance index (dynes.sec/cm^5^/m^2^)** | | | |
| Baseline (T0) | 1541.04 ± 514.33 | 1797.47 ± 563.43 | 0.100 |
| Postintubation (T1) | 1521.17 ± 466.44 | 1964.86 ± 829.14 | 0.029* |
| First surgical incision (T2) | 1718.21 ± 531.61^†^ | 2070.34 ± 617.98^†^ | 0.026* |
| Reperfusion (T13) | 1455.95 ± 429.49 | 1658.26 ± 545.81 | 0.149 |
| 15 minutes after reperfusion (T14) | 1575.30 ± 549.24 | 1687.13 ± 519.75 | 0.391 |

*Unpaired t-test, † Paired t-test compared with baseline, p < 0.05 is significant.
